# Baihu Jia Renshen Decoction for type 2 diabetic mellitus: A protocol for systematic review and meta-analysis

**DOI:** 10.1097/MD.0000000000020210

**Published:** 2020-05-08

**Authors:** Yuan Tian, Wen Zhong, Yuan Zhang, Linyue Zhou, Xiaoxu Fu, Lizhen Wang, Yan Yang, Chunguang Xie

**Affiliations:** Hospital of Chengdu University of Traditional Chinese Medicine, Chengdu, Sichuan Province, China.

**Keywords:** Baihu Jia Renshen Decoction, protocol, systematic review, type 2 diabetes mellitus

## Abstract

**Background:**

Diabetes mellitus (DM) is one of the main health problems that perplex people all over the world. The prevalence of DM is still increasing in spite of the great efforts have been made to control DM in recent years. Type 2 diabetes mellitus (T2DM) is the most common type of diabetes, accounting for about 90% of all DM cases. Traditional Chinese medicine has been used on treatment of DM and diabetic complications in China for many years. Baihu Jia Renshen Decoction (BJRD) is one of the oldest classic prescriptions of traditional Chinese medicine that applied in the field of diabetes’ treatment. BJRD is proved to be effective after years of clinical practice and basic research. The application of BJRD improves the overall clinical efficacy of T2DM. Therefore, a systematic review is necessary to provide available evidence for BJRD in therapy of T2DM.

**Methods:**

Different studies from various databases will be involved in this study. Only randomized controlled trials of T2DM patients diagnosed with World Health Organization 1999 diagnostic criteria will be included. We will search the literature in the databases from China Conference Paper Database, manual searching. Electronic database includes PubMed, Embase, Cochrane Library, Web of Science, CNKI (China National Knowledge Internet), WanFang, VIP (Chongqing VIP), and CBM (China Biomedical Literature CDROM Database). The primary outcomes include 2 hour plasma glucose, fasting plasma glucose, hemoglobin A1c, homeostasis model assessment of insulin resistance, and fasting plasma insulin. The secondary outcomes include clinical efficacy and adverse events. Data will be extracted by 2 researchers independently, risk of bias of the meta-analysis will be evaluated based on the Cochrane Handbook for Systematic Reviews of Interventions. All data analysis will be conducted by data statistics software Review Manager V.5.3. and Stata V.12.0.

**Results:**

This study will synthesize and provide high-quality evidence based on the data of the currently published BJRD for the treatment of T2DM, in terms of 2 hour plasma glucose, fasting plasma glucose, hemoglobin A1c, homeostasis model assessment of insulin resistance and fasting plasma insulin, clinical efficacy, and safety.

**Conclusion:**

This systematic review aims to evaluate the benefits and harms of BJRD for the treatment of T2DM reported in randomized controlled trials, and provide more options for clinicians and patients to treat T2DM.

**Registration number:**

INPLASY202040006.

## 1 Introduction

### 1.1 Description of the condition

Diabetes mellitus (DM) is one of the main health problems that perplex people all over the world. According to statistics reported by the World Health Organization (WHO), diabetes is the seventh leading cause of death in the world.^[1]^ The prevalence of DM is still increasing in spite of the great efforts have been made to control DM in recent years.^[2–4]^ The International Diabetes Federation reported that there are 425 million adults suffering from DM worldwide, and it was estimated that the number will reach 629 million by 2045.^[2]^ Type 2 diabetes mellitus (T2DM) is the most common type of diabetes, accounting for about 90% of all DM cases. Statistics show that the number of patients with T2DM is increasing in every country, but the low- and middle-income countries are the most affected, accounting for 77% of the total number of patients with diabetes. The situation is further worsened by the undiagnosed diabetes (fasting plasma glucose, >126 mg/dL) which affects approximately 179 million people worldwide.^[5]^

### 1.2 Description of the intervention and how it might work

In recent years, the advantages of traditional Chinese medicine (TCM) in the prevention and treatment of this kind of chronic diseases have been widely recognized around the world.^[6]^ The prescription of Baihu Jia Renshen Decoction (BJRD), first recorded in the book of *Shanghan Lun* written by Zhang Zhongjing, is composed of *Gypsum (Shigao), rhizoma anemarrhenae (Zhimu), polished round-grained rice (Jingmi), glycyrrhiza (Gancao)and Ginseng (Renshen)*. It is recorded in the book that BJRD can be used to treat diseases with the main symptoms of fever, sweating, thirst, and great pulse. The above symptoms are also the main syndromes of *Xiaoke syndrome. Xiaoke syndrome* is DM in TCM. The basic pathogenesis of diabetes in TCM is dryness-heat due to deficiency of yin.^[7]^ BJRD contraposes this pathogenesis, clears away heat and moistens dryness, nourishes yin and promotes fluid production. Therefore, BJSD has been widely used in the clinical treatment of diabetes and achieved good results.^[8–11]^

In addition, pharmacological studies have shown that *rhizoma anemarrhenae* is rich in hypoglycemic chemicals such as anemarrhena alcohols and anemarrhena saponins, and so on.^[12–16]^ Ingredients such as ginsenosides in ginseng can also lower blood sugar and improve insulin resistance.^[17–19]^

### 1.3 Why it is important to this review

BJRD is frequently used clinically in DM,^[8–11]^ especially as a complementary therapy. The application of BJRD proves to be more effective in the treatment of T2DM than using routine treatment only.^[11,20,21]^ The latest meta-analysis^[22]^ was published in 2016, retrieved articles before January 1st, 2016. However, this article only included 12 studies, and these included articles were generally of low quality, small sample size, and unclear random grouping. After 4 more years, a new meta-analysis is necessary in to achieve trustworthy evidence.

### 1.4 Objectives

This review aims to systematically evaluate the benefits and harms of BJRD for T2DM patients reported in randomized clinical trials (RCTs). We look forward to provide more reliable evidence on as a supplementary treatment, does BJRD really improve the efficacy of treatment on T2DM.

## 2 Methods

### 2.1 Protocol registration

The protocol of the systematic review has been registered in INPLASY.COM, and the registration number is INPLASY202040006. This systematic review protocol will be conducted and reported strictly according to preferred reporting items for systematic reviews and meta-analyses^[23]^ statement guidelines, and the important protocol amendments will be documented in the full review.

### 2.2 Inclusion criteria

#### 2.2.1 Study design

Only RCTs (except Quasi-RCTs and cluster RCTs) will be included. Animal mechanism studies and non-RCTs will be excluded. Article that substantially overlaps with another published article in print or electronic media will be excluded. Duplicate publications produced by a single experiment and published as separate papers with different criteria for measuring results, priority will be given to original publications and other publications will be excluded. The language and time of publication will not be restricted.

#### 2.2.2 Participants

The patients of type 2 diabetes (using WHO 1999 diagnostic criteria^[24]^). These types of patients will not be included: patients with acute complications of diabetes; patients with severe heart disease, liver and kidney dysfunction, mental illness, or a relevant drug allergic history and patients during pregnancy or lactation.

#### 2.2.3 Interventions

Both groups were cured with conventional diabetes treatments recommended by the American Diabetes Association (ADA) guidelines, including diet, exercise, and hypoglycemic and lipid-lowering therapies.^[25]^ The experiment group used BJRD or modified BJRD, while the control group applied for placebo or no treatment. In addition, the 2 groups did not take any drugs that interfered with the outcome indicators.

#### 2.2.4 Outcomes

The primary outcomes include 2 hour plasma glucose, fasting plasma glucose, hemoglobin A1c, homeostasis model assessment of insulin resistance, and fasting plasma insulin.

The secondary outcomes include clinical efficacy and adverse events. The clinical efficacy refers to the guiding principles for clinical research of new Chinese medicines^[26]^ and is determined according to the degree of improvement of the symptoms of the patient before and after treatment: markedly effective: symptoms improved significantly more than 70%; effective: symptoms reduced by 30% to 70%; ineffective: symptom improvement is less than 30% or no improvement, or even worse.

It is worthy of note that clinical efficacy evaluation in the guiding principles for clinical research of new Chinese medicines is determined according to the degree of improvement of the symptoms of the patient before and after treatment, so the evaluation of clinical efficacy might be relatively subjective. However, this study insists on this evaluation method because clinical symptoms are the key basis of TCM in diagnosis and evaluation of curative effect.

### 2.3 Search methods

#### 2.3.1 Search resources

This review will include grey literature sourced from China Conference Paper Database, manual searching. Electronic database includes PubMed, Embase, Cochrane Library, Web of Science, CNKI, WanFang, VIP, and CBM will also be searched. We will simply present the search process of the PubMed (Table 1). The data will be searched in English and Chinese databases from their inception to April 2020.

**Table 1:**
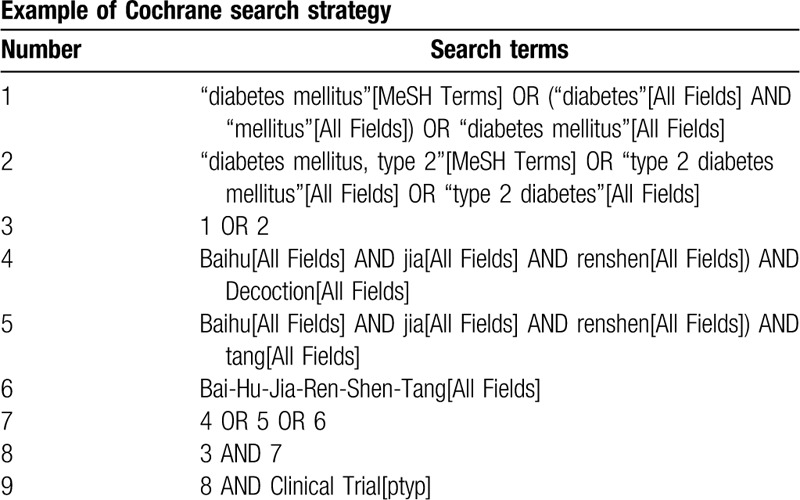
PubMed search strategy.

#### 2.3.2 Search strategies

The following MeSH terms and their combinations will be searched:

Baihu Jia Renshen Decoction OR Baihu Jia Renshen Tang;

Diabetes mellitus, type2 OR Diabetes mellitus;

Clinical trials.

### 2.4 Data collection and analysis

#### 2.4.1 Studies selection

**Figure 1:**
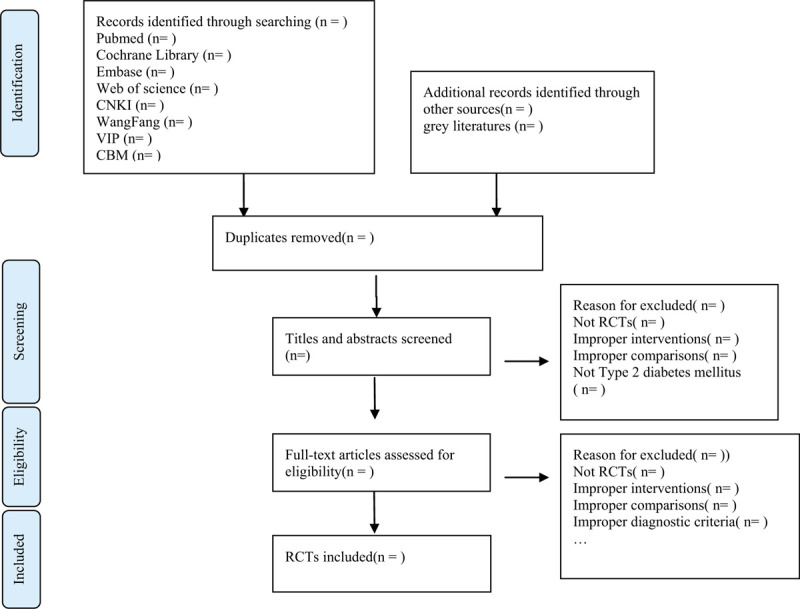
Flow chart of the study selection.

There will be 2 researchers carry out the selection of research literature independently using endnote x9 software. We will first make the preliminary selection by screening titles and abstracts. Second, we will download full text of the relevant studies for further selection according to the inclusion criteria. If there is any different opinion, 2 researchers will discuss and reach an agreement. If a consensus could not be reached, there will be a third researcher who make the final decision. Details of the selection process were shown in the flow chart (Fig. 1).

#### 2.4.2 Data extraction

Two researchers will read all the included text in full, and independently extract the following information:

(1) basic information (year of publication, the first authors name),
(2) study method (design, blinding),
(3) information of participants (number of patients, gender, age, course of disease),
(4) information of treatment (interventions and controls, medicine, dose, frequency, duration),
(5) outcomes (2 hour plasma glucose, fasting plasma glucose, hemoglobin A1c, homeostasis model assessment of insulin resistance, fasting plasma insulin, clinical efficacy, adverse effect),
(6) *P*-value.

If we could not reach an agreement, a third researcher would make the final decision. One researcher would contact the corresponding author by telephone or e-mail for more information when the reported data were insufficient or ambiguous.

#### 2.4.3 Assessment of risk of bias

All the included studies will be evaluated based on the guidelines of Cochrane Handbook for Systematic Reviews of Interventions.^[27]^ The quality of each trial will be categorized into “low,” “unclear,” or “high” risk of bias according to the following items: adequacy of generation of the allocation sequence, allocation concealment, blinding of participants and personal, blinding of outcome assessors, incomplete outcome data, selected reporting the results and other sources of bias (such as comparable baseline characteristic, inclusion and exclusion criteria).

#### 2.4.4 Assessment of reporting biases

Reporting biases and small-study effects will be detected by funnel plot and Egger test if there are 10 more studies included in this Meta-analysis. For Egger test, *P*-value of <0.10 was considered to indicate the exist of reporting biases and small study effects.

#### 2.4.5 Data analysis

We used Revman 5.3 software provided by the Cochrane collaboration to analyze the data. Binary outcomes will be summarized using risk ratio with 95% confidence interval for relative effect. Continuous outcomes will be summarized by using weighted mean difference with 95% confidence interval. We will use random-effect model for meta-analysis in this review according to research recommendations.^[28]^

Statistical heterogeneity will be assessed by *X*^2^ and *I*^2^ statistical tests. Where *P*-value ≥ .1 and *I*^2^ ≤ 50%, there is no obvious statistical heterogeneity among the studies. On the contrary, where *P*-value < .1 or *I*^2^ > 50% indicates a considerable heterogeneity. Meta-analysis will be performed when the statistical heterogeneity is acceptable (*P*-value ≥ .1 and *I*^2^ ≤ 50%), otherwise, subgroup analysis will be applied to explore the influence of potential factors on the outcome measures. We will conduct subgroup analyses by different race, age, gender, course of treatment, and different type of BJRD (intervention forms, pharmaceutical dosage form, dosage, etc). We will conduct sensitivity analyses by omitting studies one by one to probe the impact of an individual study. If a meta-analysis cannot be performed, we will conduct descriptive analysis instead.

#### 2.4.6 Patient and public involvement

This is a meta-analysis study based on previously published data, so patient and public involvement will not be included in this study.

#### 2.4.7 Ethics and dissemination

Ethical approval will not be required as this is a protocol for systematic review and meta-analysis. The findings of this study will be disseminated to a peer-reviewed journal and presented at a relevant conference.

#### 2.4.8 Evidence assessed

The quality of evidence for this study will be assessed by “grades of recommendations assessment, development, and evaluation (GRADE)” standard established by the WHO and international organizations.^[29]^ To achieve transparency and simplification, the quality of evidence is divided into 4 levels in GRADE system: high, medium, low, and very low. We will employ GRADE profiler 3.2 for analysis.

## 3 Discussion

DM is one of the main health problems that perplex people worldwide.^[1]^ Although the research in the field of T2DM has never been interrupted and the treatment methods are increasingly mature, the incidence of T2DM is still growing.

TCM has been used on treatment of DM and diabetic complications in China for many years.^[30,31]^ BJRD is one of the oldest classic prescriptions of TCM that applied in the field of diabetes’ treatment. As early as 1974, animal experiments already showed that BJRD significantly reduced blood sugar in diabetic mice.^[9]^ At the later stage, evidences show that both the BJRD compound^[32]^ and the single herb^[12–19]^ that constitutes the BJRD prescription have a clear therapeutic effect on diabetes. The application of BJRD improves the overall clinical efficacy of T2DM.^[11,20,21]^

In this study, we attempt to perform an updated meta-analysis to provide high-quality evidence for the clinical efficacy and safety of BJRD. We hope this study will provide more options for clinicians and patients to treat T2DM. However, there might be some potential deficiencies in this systematic review. First, the clinical trial design of TCM we intend to include is difficult to achieve single- or double-blind method throughout all the research. Second, the variety of race, age, gender, intervention forms, pharmaceutical dosage form, dosage, and treatment course may result in higher clinical and statistical heterogeneity.

## Author contributions

**Conceptualization:** Yuan Tian, Wen Zhong.

**Data curation:** Yuan Zhang, Linyue Zhou, Lizhen Wang.

**Formal analysis:** Yuan Zhang, Linyue Zhou.

**Methodology:** Yan Yang, Yuan Zhang, Xiaoxu Fu.

**Project administration:** Chunguang Xie.

**Resources:** Yuan Tian, Wen Zhong, Chunguang Xie.

**Software:** Yaun Tain, Wen Zhong, Chunguang Xie.

**Supervision:** Wen Zhong, Chunguang Xie.

**Writing – original draft:** Yuan Tian.

**Writing – review and editing:** Chunguang Xie.
